# Development of Middle Stone Age innovation linked to rapid climate change

**DOI:** 10.1038/ncomms2897

**Published:** 2013-05-21

**Authors:** Martin Ziegler, Margit H. Simon, Ian R. Hall, Stephen Barker, Chris Stringer, Rainer Zahn

**Affiliations:** 1 School of Earth and Ocean Sciences, Cardiff University, CF10 3AT Cardiff, UK; 2 Department of Earth Sciences, Natural History Museum, SW7 5BD London, UK; 3 Institució Catalana de Recerca i Estudis Avançats (ICREA), 08010 Barcelona, Spain; 4 Institut de Ciència i Tecnologia Ambientals (ICTA), Universitat Autònoma de Barcelona, 08193 Bellaterra, Spain; 5 Present address: Geological Institute, ETH Zürich, 8092 Zürich, Switzerland

## Abstract

The development of modernity in early human populations has been linked to pulsed phases of technological and behavioural innovation within the Middle Stone Age of South Africa. However, the trigger for these intermittent pulses of technological innovation is an enigma. Here we show that, contrary to some previous studies, the occurrence of innovation was tightly linked to abrupt climate change. Major innovational pulses occurred at times when South African climate changed rapidly towards more humid conditions, while northern sub-Saharan Africa experienced widespread droughts, as the Northern Hemisphere entered phases of extreme cooling. These millennial-scale teleconnections resulted from the bipolar seesaw behaviour of the Atlantic Ocean related to changes in the ocean circulation. These conditions led to humid pulses in South Africa and potentially to the creation of favourable environmental conditions. This strongly implies that innovational pulses of early modern human behaviour were climatically influenced and linked to the adoption of refugia.

## Abstract (web summary)

The South African archaeological record contains evidence of the early flourishing of the human mind. Ziegler *et al*. provide new paleoclimate reconstructions, which suggest that rapid fluctuations in global climate have played a key role in the evolution of these early human cultures.

## Graphical abstract

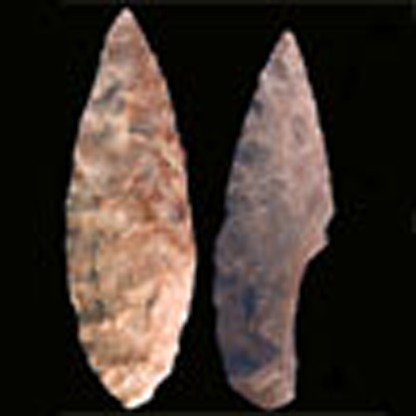

Archaeological and genetic evidence suggest that anatomically modern humans (the modern form of *Homo sapiens*) originated in Africa during the Middle Stone Age (MSA), which lasted from about 280,000–30,000 years ago^1^,^2^, although fossil and genetic data are ambivalent about specific areas of origin^2^. There has also been considerable debate about the factors behind cultural evolution in general, and the emergence of modern human behaviours in particular^3^. By analogy with the forces driving biological evolution, cultural change might, for example, have largely been caused by random factors such as drift^4^, or driven by adverse conditions^5^, or be due to increases in population density and networking^6^.

Excavations in South African archaeological sites (Fig. 1) and new developments in dating techniques place the South African MSA industries in a very well-constrained temporal context^7^,^8^,^9^,^10^,^11^,^12^. These studies document several abrupt pulses of major technological advancement that have been interpreted to reflect the emergence of modern behaviours of innovation, language and cultural identity^7^,^11^. Among the most important periods are the South African Still Bay (SB) and Howiesons Poort (HP) industries that are dated to ∼71,500 and 64,000–59,000 years ago and are widely viewed as dynamic periods of MSA innovation. Examples include symbolic expression through engraved ochres, stone and bone tools, shell jewellery and plant bedding constructions^7^,^10^,^12^. These periods of innovation appear to be unique and as such they may have been one of the forces behind the growth and dispersal of human populations within and out of Africa^2^,^13^. However, the causes behind the timing of the seemingly irregular appearance, punctuated occupational episodes and sudden disappearance of these industries in South Africa are poorly understood. While it has been speculated that there may have been some impact of local- to regional-scale environmental variations^8^,^14^, it has also been argued that climate change had little part in the development of MSA industries^7^.

A diverse range of climates characterizes modern South Africa, including the Mediterranean climate of the Western Cape, (semi-) desert conditions of the West Coast and the subtropical climate in the eastern part. There is a high degree of interannual rainfall variability, which impacts greatly on water resources, agriculture and rural communities. Regional climate in the Eastern Cape is dominated by austral summer rainfall, primarily dictated by the seasonal interplay between subtropical high-pressure cells and the migration of easterly flows associated with the Intertropical Convergence Zone (ITCZ) that brings rain to the tropics (Fig. 2). The region becomes cool and dry during the austral winter months as the land surface cools relative to the oceans and a broad anticyclonic circulation prevails.

Recent studies have improved our general understanding of the climatic mechanisms governing hydrological changes in tropical and subtropical sub-Saharan Africa over the Pleistocene and suggest that recurrent latitudinal shifts of the ITCZ acted as a primary forcing factor of climate change on these timescales^15^,^16^,^17^. During the last million years or so, global climate has repeatedly alternated between interglacial (akin to pre-industrial conditions) and glacial states (with large continental ice sheets in the Northern Hemisphere) with an average pacing of ∼100 kyr through the combined influences of orbital precession and obliquity on insolation^18^. Tropical climate and its monsoonal systems follow a different pacing (23 kyr), due to the dominance of orbital precession in driving low-latitude summer insolation^19^,^20^,^21^. Stronger summer insolation intensifies atmospheric convection and in consequence leads to higher rainfall. Such a link between precession forcing and long-term changes in monsoonal climates is supported by climate modelling studies^19^,^22^. As a consequence of the precessional forcing, boreal and austral summer insolations vary out of phase and the average position of the ITCZ migrates latitudinally^20^,^23^.

In addition to orbital-scale variability, global climate over the last few glacial cycles has been punctuated by changes on much shorter timescales^24^. These millennial-scale climate fluctuations are characterized by abrupt (often within decades) and large (up to 10 °C in mean annual temperatures) changes in Northern Hemisphere high-latitude temperatures, as recorded in Greenland ice-core records^25^ and out-of-phase but equally abrupt temperature changes in the South Atlantic Ocean^26^ with more gradual changes over Antarctica^27^. This bipolar seesaw behaviour has been linked to changes in the strength of the Atlantic meridional overturning circulation and its effect on the distribution of heat between the hemispheres^28^. In response to abrupt Northern Hemisphere cooling, the annual average position of the ITCZ shifts to a more southward position, again resulting in an opposing response between the northern hemisphere, where monsoons weaken^20^,^29^ and the southern hemisphere where monsoons strengthen^30^,^31^. The presence of a large-scale atmospheric teleconnection linking Northern Hemisphere cold events and precipitation changes in sub-Saharan Africa is also consistent with climate modelling experiments^32^,^33^,^34^.

Here we present a marine record of highly variable runoff from coastal rivers to the eastern South African continental margin that reflects rainfall variability in the Eastern Cape during the MSA, and provides evidence for a direct link between abrupt climate change and the development of cultural complexity in early humans.

## Results

### High-resolution record of river discharge in SE Africa

**Figure 1:**
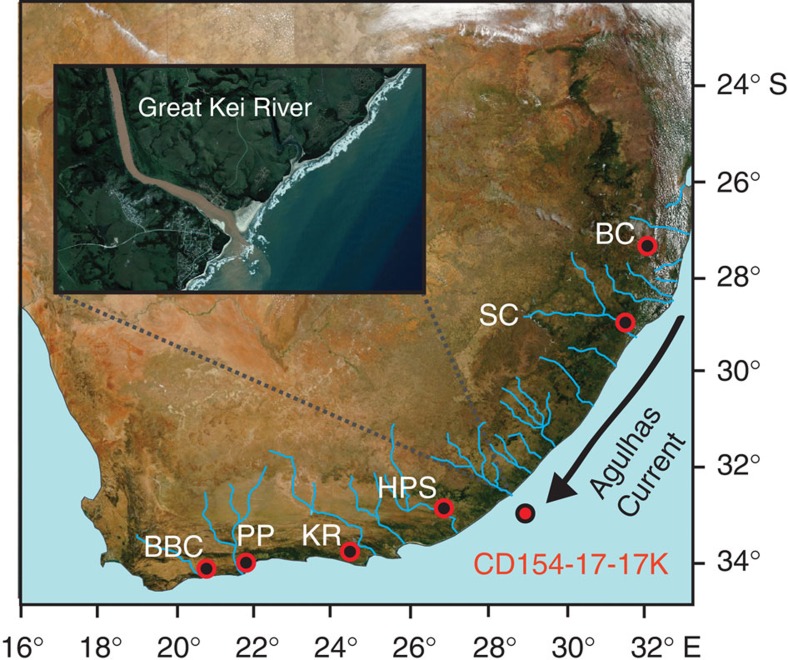
Location of studied sediment core and archaeological sites in South Africa. Archaeological sites are: Blombos Cave (BBC), Pinnacle Point (PP), Klasies river (KR), Howiesons Poort Shelter (HPS), Sibudu Cave (SC) and Border Cave (BC). Inset shows satellite image of the Great Kei river mouth, with its typical brownish-red river water derived from a high, iron-rich, sediment load.

Marine sediment core CD154-17-17K (33° 16.13′ S, 029° 07.29′ E, 3,333 m water depth) was retrieved from ∼95 km off the Eastern Cape coast near the mouth of the Great Kei river (Fig. 1). The initial chronology of the core is established through eight radiocarbon dates in the upper part of the core and graphically correlating the planktonic foraminiferal (*Globigerinoides ruber*) oxygen isotope (*δ*^18^O) record to the Antarctic deuterium (temperature) record of the EPICA Dome C ice core^35^. The planktonic *δ*^18^O record reflects the combined influence of ambient sea-surface temperature variability in the Agulhas Current and global ice volume changes and shows a good fit with the long-term temperature variability in Antarctica (Fig. 3). The sediment core spans a time period of approximately the last 100,000 years with an average sedimentation rate of 4 cm kyr^−1^. Elemental concentrations across the whole core were obtained with an X-ray fluorescence (XRF) core scanner (ITRAX). The relative element intensity counts obtained from the XRF scanning were calibrated to concentrations using a suite of individual samples analysed for absolute bulk elemental composition. The major oxide elemental ratios in sediments from CD154-17-17K are very similar to the ratios in the suspended load of rivers in South Africa^36^ that drain similar rock types as the Great Kei river, suggesting that the terrestrial material is of local origin. The most proximal source for terrestrial material to CD154-17-17K is the Great Kei river, which is ∼520 km long and has a catchment area of 20,566 km^2^, forming the southern border of the Transkei coast of the Eastern Cape (Fig. 1). Several other rivers also enter the Indian Ocean to the north of our core site. These include the Mbashe, Umzimvubu and Umtata rivers, as well as the Tugela, the largest in the KwaZulu-Natal Province. These rivers are all typical brown-water rivers, characterized by high sediment loads. Their sediments, in particular those derived from the latosol-type soils, derived from mudstones and sandstones of the Karoo Supergroup and associated intruded dolerites (‘Ironstone') within the catchment areas, are notably rich in iron (Fe) oxides. Consequently, the Fe/Ca ratio recorded in CD154-17-17K can be used as a first-order indication of relative changes in the amount of fine (Fe-rich) terrigenous components supplied to the core site from regional river discharge. However, we use the Iron/Potassium (Fe/K) ratio as a more reliable proxy, as it is independent of possible variations in biogenic carbonate input. Fe/K ratios serve as indicator of changes between humid and dry conditions^34^,^37^. Govin *et al*.^37^ demonstrate, that the spatial distributions of Fe/K in marine core-top samples reflect the relative input of intensively weathered material from humid regions versus slightly weathered particles from drier areas. In tropical humid regions, high precipitation promotes intense chemical weathering of bedrocks^38^, resulting in highly weathered soils whose geochemical signature, rich in Fe, is transferred to marine sediments by fluvial input. In contrast, K derives from potassium feldspar or illite, which are both characteristic of drier regions with low chemical weathering rates^39^. Govin *et al*.^37^ find that low Fe/K values indicate dominant deposition of only slightly weathered particles originating from relatively dry areas on the subtropical African margin. Conversely, suspended material from the major river systems exhibits high Fe/K ratios. In addition, the spatial Fe/K distribution observed along the African continental margin and in African dust- and river-suspended samples reflects the spatial distribution of African soil types. High Fe/K values recorded in the tropics reflect the presence of intensively weathered soils enriched in Fe over the adjacent continent. Govin *et al*.^37^ conclude therefore, that Fe/K ratios of surface sediments can be used to reconstruct African and South American climatic zones. Fe/K ratios have also been applied as a proxy for fluvial versus aeolian input, with high values indicating an increased supply of river-suspended material relative to dust deposition^34^. Together, these proxies provide a high-resolution record of variable hydrological conditions in the Eastern Cape.

### Millennial-scale climate variability in the Eastern Cape

The Fe/Ca and Fe/K records are prominently punctuated by a series of abrupt events, indicating pulses in river input and more humid conditions (Fig. 3). Within the uncertainty of our age model, it is possible to link each individual event in the Fe/K record to a corresponding millennial-scale cold event in the Northern Hemisphere temperature as documented in Greenland Ice core records^25^. The amplitude of the long-term variability appears to be larger in the Fe/Ca record than in the Fe/K ratio, which may indicate that variable carbonate production and preservation may have an additional role on these longer timescales. Sea-level changes may have had an impact as well, with increased valley river incision during sea-level low stands and, in consequence, a larger discharge of terrigenous sediments. However, these processes have no effect on the ratio of the terrigenous elements Fe and K.

Modelling studies confirm that remote atmospheric forcing during Northern Hemisphere cold events is a key driver of hydrological variability in South Africa, resulting in wetter conditions during these events^40^,^41^,^42^. A mean southeastward shift in the positions of the South Indian and South Atlantic Ocean anticyclones would lead to increased rainfall over the Eastern Cape. In contrast, palaeoclimate reconstructions from Lake Malawi^15^, Lake Tanganyika^43^ and the Sahel zone^34^ indicate much dryer conditions associated with Northern Hemisphere cold events, reflecting the southward shift of the ITCZ during these periods. Similarly, West Africa experienced weaker monsoonal precipitation during these events^29^. Evidence from an ocean–atmosphere general circulation model^44^ suggests that this linkage also occurs because a weakened meridional overturning circulation in the Atlantic Ocean leads to a bipolar seesaw warming response in the equatorial South Atlantic Ocean^28^, and consequently a reduction in West African summer monsoonal winds and rainfall over West Africa^29^. While large parts of sub-Saharan Africa faced severe dry conditions during North Atlantic cold events^45^, South Africa apparently experienced more humid conditions. Precipitation in the Eastern Cape is strongly dependent on the Agulhas Current sea-surface temperatures, with a warm current providing supply of low-level moisture and buoyancy to facilitate the occurrence of deep convection and rainfall, as the onshore flow reaches the coast^46^. Associated with the bipolar seesaw response, the Southern Ocean experiences a southward shift of the subtropical front during North Atlantic cold events^26^, and a southward shift of the southern Hemisphere Westerlies is associated with an increase in the wind stress curl in the South Indian Ocean and warming in the Agulhas Current^47^.

The East Asian summer monsoon also responded sensitively to these glacial cooling events in the Northern Hemisphere. Oxygen isotope records in speleothems from Chinese Caves provide a high-resolution and precisely dated (U/Th) record of East Asian–Indian Monsoon intensity^20^,^48^,^49^ and indicate weakened monsoon intensity during Northern Hemisphere cold events. When compared directly, our record of Eastern Cape riverine input (humid versus dry conditions) and the Chinese speleothem record show a remarkable similarity in the structure of millennial-scale events (Fig. 3) that also fit dynamically with the interhemispheric signal propagation in that they indicate opposite wet–dry successions between the records. We take advantage of the precise absolute dating of the speleothem record to further fine-tune the age-scale of CD154-17-17K by synchronizing transitions into and out of the millennial excursions in both records. The average age difference between the initial and this fine-tuned age model is only 0.07 kyr. Absolute age differences for individual tie points are generally within 1 kyr and always less than ±1.8 kyr and are therefore well within the age uncertainty of the initial low-resolution planktonic foraminiferal *δ*^18^O-based age model. We adopt the speleothem-based ages in the following detailed comparison of millennial-scale climate events with the archaeological record and the dates of well-documented phases in behavioural and technological innovation such as the SB and HP industries of the MSA^7^.

## Discussion

Many different views have developed about possible links between climate change and the appearance or disappearance of MSA industries such as the SB and HP in southern Africa. Early work speculated that the HP was the result of changes in the adaptation of an indigenous population in response to environmental change^50^. At the time, age control of the archaeological record, as well as the palaeenvironmental records, was not sufficient to test this hypothesis. It was later also argued that changes in the Earth's orbital configuration led to the development of relatively cool and wet conditions in southern Africa during Marine Isotope Stage (MIS) 4 (ref. 51), while speleothem deposits recovered at Pinnacle Point Cave on the southern coast suggest variable conditions prevailed at this time^14^, broadly coeval with the SB and HP industries. It has also been argued that rather than catastrophic events, variability associated with spatially and temporally complex climate conditions is a significant factor in itself^52^. Recently, McCall and Thomas^53^ have taken this view further by proposing that the mobility patterns and organizational characteristics of the SB may have been part of a strategy for dealing with environmental unpredictability and short-term climate fluctuations that occurred during this interval. They argued that human populations were relatively large following expansion during the benign conditions of MIS 5, but the SB and HP industries followed as a stress response to the colder and more variable conditions of MIS 4. There have also been arguments for the origins of the SB and HP industries in demographic terms as bursts of population increase and consequent higher archaeological visibility^54^.

We find a striking correspondence between the archaeological record of South Africa and the timing of abrupt climate change as inferred from our CD154-17-17 marine palaeo-record on the chronology of the absolutely dated speleothem record (Fig. 4). Major events of occupation in archaeological sites coincide consistently with North Atlantic cold events, weakened Asian summer monsoon periods and inferred increased river discharge and humid conditions in the Eastern Cape. The age of the SB industry (71.9–71.0 ka (ref. 7)) coincides within the error margins with one of the most extreme cold events in the Northern Hemisphere (cold Greenland stadial 19), a period of a particularly weak Asian Monsoon lasting from ∼72.8 to 71.5 ka. An earlier settlement (early SB) with plant bedding construction at Sibudu Cave (∼77 ka) (ref. 12) can be also linked to one of the first abrupt North Atlantic cold events (cold Greenland stadial 20) at the MIS 5/4 transition (77.1–76.4 ka). Similarly, the duration of the HP industry (64.8–59.5 ka (ref. 7)) coincides with cold conditions in the North Atlantic Ocean and a dry phase in Asia. Our record from core CD154-17-17K suggests particularly wet (humid) conditions in the Eastern Cape during each of these intervals. The abrupt ending of the HP industry at ∼59.5 ka coincides with a rapid transition to drier conditions. During this transition, the Northern Hemisphere warmed abruptly (into MIS 3) and summer monsoon strength in West Africa and Asia increased. MSA events and occupation of the major sites during MIS 3 are rare. This may be explained by generally drier conditions in South Africa, whereas improved conditions in East Africa may have shifted the main focus of demographic expansion and innovation to that region. However, three well-dated occupational pulses at Sibudu Cave, which have been named as post-HP (58.5+/−1.4 ka), late MSA (47.7+/−1.4 ka) and final MSA (38.6+/−1.9 ka (ref. 8)) again correlate well with abrupt Northern Hemisphere cold events (Heinrich events). Our marine sediment core again indicates again short pulses of wetter conditions in the Eastern Cape. Our findings are supported by evidence from Sibudu Cave, where sediments that have built up during occupational periods indicate relatively moist conditions^55^.

Dates of MSA events predating the SB and HP industries are rare and have larger age uncertainties. The intermittent human occupation at Pinnacle Point Cave, showing evidence for the utilization of marine resources as well as the production of stone tools^11^, has been linked to sea-level changes^56^. Some of these dates at Pinnacle Point are greater than the oldest samples from our record. However, the Chinese speleothem record, which may be used as a template for the interhemispheric teleconnection that we observe, indicates a particularly weak East Asian monsoon around the older occupational phases at ∼120 ka and 165 ka, and hence we can infer that these settlements probably coincided with wetter conditions in South Africa. The date of a 100,000-year-old ochre workshop at Blombos Cave has a relatively large uncertainty^9^ but appears to fall within a period of wet conditions in SE Africa during the precession maximum of MIS5b. Comparing the dates of occupational phases during the Later Stone Age with the Fe record again corroborates the overall relationship. One example is a period of limited archaeological Later Stone Age evidence in South Africa that dates between 24 and 16 ka (ref. 57), and which coincides with a longer period of continuously dry conditions in South Africa during the last glacial maximum.

The remarkable correlation between the millennial-scale Northern Hemisphere cold events (independently dated in the Chinese speleothem record) and the documented archaeological evidence on land for pulsed phases of human expansion and technological innovation suggests that the well-known major progressions in the development of modern humans during the MSA can be linked with intervals of abrupt climate change. A major control on survival for any human population is access to fresh water supply. Abrupt Northern Hemisphere cooling events and the associated major shifts in tropical climate dynamics led to extended droughts in large parts of the African continent, which potentially repeatedly bottlenecked early human populations elsewhere. However, conversely, the same abrupt cooling also created favourable humid ‘refugial' conditions in southern Africa, which along with the highly diverse vegetation (Cape Floral Kingdom) and a rich coastal ecosystem, would have combined to provide ample resources for early human expansion^56^. The resultant demographic pulses can be linked to the innovations of the SB and HP industries, thus supporting one of the key models of cultural change in the Palaeolithic—a correlation between innovation and the adoption of new refugia with subsequent increases in population size, density and both intra- and inter-group networking^6^. Moreover, such climate-driven pulses in southern Africa and more widely were probably fundamental to the origin of key elements of modern human behaviour in Africa, and to the subsequent dispersal of *Homo sapiens* from its ancestral homeland^2^. Our record of recurrent moist humid episodes serves as a template for future archaeological work to assess if the connection with abrupt climate change extends to other as yet less-well-documented innovation pulses in Africa.

## Methods

### Planktonic oxygen isotopes

Surface-dwelling planktonic foraminiferal species *Globigerinoides ruber* was picked from the 250–315 μm size fraction for oxygen isotope (*δ*^18^O) analyses. At least 25 specimen for each sample were analysed. All the analyses were performed using a Thermo Scientific Delta V Advantage mass spectrometer with an automated carbonate preparation device (GasBench III) at the Cardiff University stable isotope laboratory. Stable isotope results were calibrated to the PDB scale by international standard NBS19. The analytical precision is better than ±0.1%.

### Bulk elemental concentrations

XRF core scanning allows analysis of the chemical composition of marine sediments directly at the surface of a split sediment core. The XRF core scanning technique is nondestructive, nearly continuous and comparably fast. The chemical composition of the sediment is measured in element intensities in total counts, which are proportional to the chemical concentrations. A detailed description of the scanning methodology can be found in Croudace *et al*.^58^. Core scanning was performed using the ITRAX XRF Core scanner at the British Ocean Core Research Facility (BOSCORF, Southampton). Measurements were made at 1 cm resolution with a count time of 30 s, at 30 kV and 50 mA on the X-ray tube.

Additionally to the XRF scanning, major element and trace elements were analysed for a subset of samples. This subset of samples has been used to calibrate the XRF scanning counts (Fig. 5). Analysis was performed by a Thermo X Series 2 inductively coupled plasma mass spectrometer (ICP-MS). Approximately 2 g of freeze-dried and ground sediment was ignited in a furnace at 900 ^°^C (58) loss on ignition values. Whole-sediment major element concentrations were obtained following Li metaborate fusion^59^. The internationally recognized standard JB-1A was run alongside the sample batch. Long-term relative standard deviations show precision of 1–2% for major trace elements for JB-1A.

### Age model

**Figure 2:**
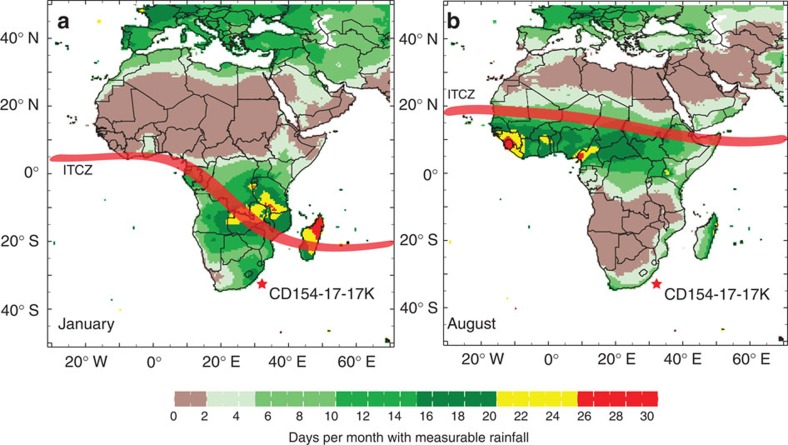
Modern rainfall variability over Africa. (**a**) January and (**b**) August. Colours indicate days per month with measurable rainfall. The climate over large parts of Africa is characterized by a strong seasonality with summer monsoonal rainfall and the approximate position of the ITCZ (red band) migrating between the north and south of the continent over the course of the year.

**Figure 3:**
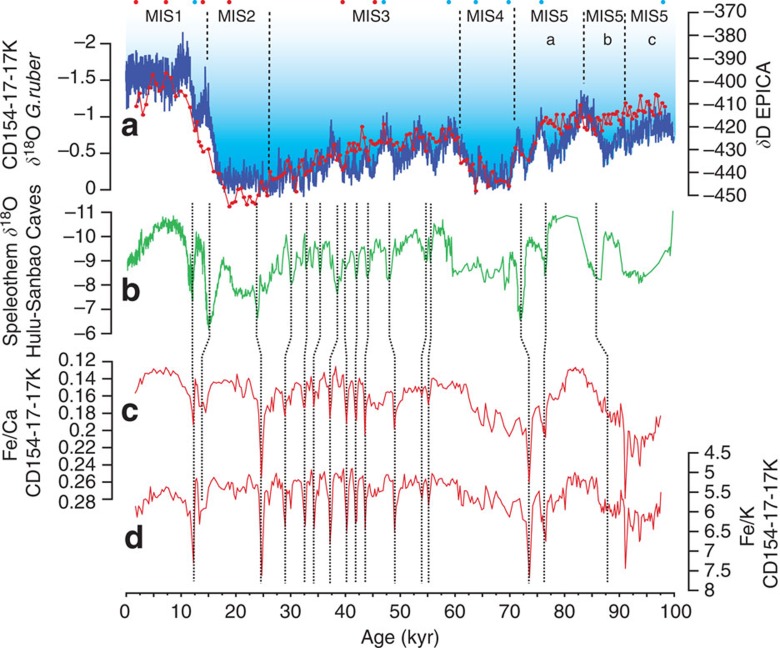
Age model construction. (**a**) Graphical correlation of *G. ruber δ*^18^O record of CD154-17-17K (red) to the EPICA Dome C deuterium record (purple) (EPICA) on speleothem-timescale of ref. 24. Age control points as dots, red dots are based on radiocarbon dates and blue dots are based on the tuning of the foraminiferal *δ*^18^O record. (**b**) *δ*^18^O splice from Chinese speleothems (green) (Hulu and Sanbao Cave)^20^,^48^. (**c**) Fe/K ratio of CD154-17-17K on the initial age model. (**d**) Fe/Ca ratio of CD154-17-17K on the initial age model. Stippled lines show the fine-tuning of the initial age model through graphical correlation of Fe/K ratio of CD154-17-17K to *δ*^18^O splice from Chinese speleothems (Hulu and Sanbao Cave).

**Figure 4:**
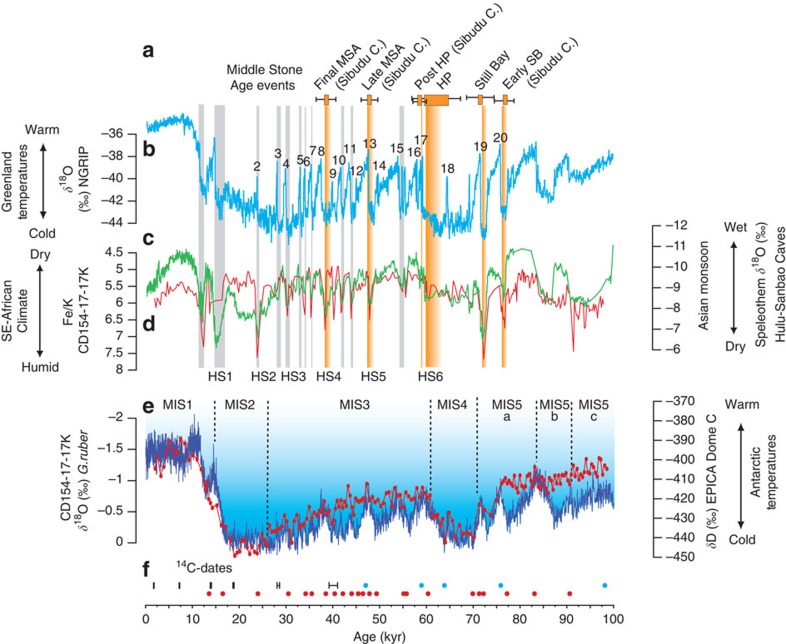
Climate change during the Middle Stone Age in Southeast Africa. (**a**) Ages of Southeast African Middle Stone Age events^7^,^8^,^10^ (error bars indicate 2-sigma ranges), coinciding with Heinrich events 4–6 and cold Greenland stadials 19 and 20. (**b**) *δ*^18^O record from Greenland ice core NGRIP^25^ (light blue, Speleo-age model presented in Barker *et al*.^24^) displaying abrupt temperature variability in the North Atlantic. Numbers indicate warmer stadials. Underlying bars indicate cold stadials with more extreme Heinrich stadials (HS1–HS6) highlighted (**c**) *δ*^18^O splice from Chinese speleothems (green) (Hulu and Sanbao Cave)^20^,^48^ showing synchronous variability of the East Asian summer monsoon with climate variability in the North Atlantic and (**d**) Fe/K of CD154-17-17K (red) indicating higher river discharge and wetter Eastern Cape Province climate during Northern Hemisphere stadials. (**e**) Planktonic foraminiferal (*G. ruber*) *δ*^18^O record from CD154-17-17K (red), reflecting global ice volume variability and local sea-surface conditions, in comparison with EPICA Dome C deuterium record (blue)^35^. Marine isotope stages (MIS) are indicated. (**f**) Age control points for CD154-17-17K, including radiocarbon dates (black), tuning of the foraminiferal *δ*^18^O record.

**Figure 5:**
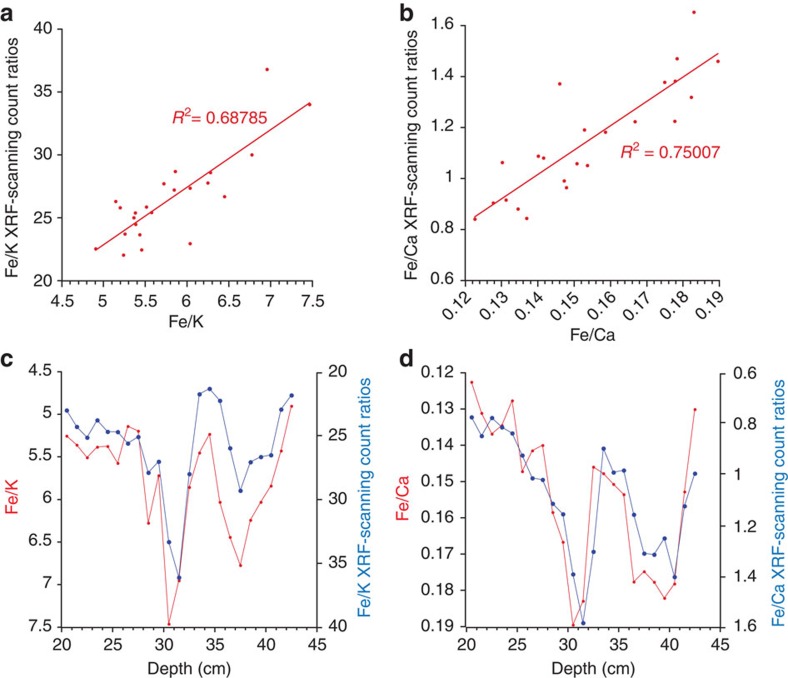
Calibration of the XRF scanning counts. Calibration using a set of samples that was analysed for absolute bulk elemental concentrations using ICP-MS. (**a**) Fe/K XRF scanning counts versus absolute Fe/K ratios. (**b**) As in **a** but for Fe/Ca. (**c**) Fe/K scanning counts and absolute Fe/K versus depth over the calibrations interval. (**d**) As in **c** but for Fe/Ca.

The chronology of core CD154-17-17K is derived from eight radiocarbon dates in the upper part of the record (Table 1) and additionally by graphical correlation of the planktonic foraminiferal (*G. ruber*) oxygen (*δ*^18^O) isotope record to the deuterium (temperature) record of Antarctic ice core EPICA Dome C^35^ on the on the Speleo-age model of Barker *et al*.^24^ (Fig. 2, Table 2). The planktonic δ^18^O record reflects the combined influences of local sea-surface temperature variability and global ice volume changes and a limited number of age control points is sufficient to achieve a very good fit with the temperature record from Antarctica. This age model shows that the sediment core spans the time period over the last ∼100,000 years with an average sedimentation rate of 4 cm kyr^−1^, ranging from 1.5–5.2 cm kyr^−1^. To further fine-tune the age model, we visually match common transitions within the Fe/K ratio and the speleothem record from Chinese Caves, Hulu^48^ and Sanbao^20^ (Fig. 3). We produced a spliced record from the following records: SB 26 (0.46–5.32 ka), SB10 (5.36–11.59 ka), H82 (11.60–14.94 ka), SB3 (14.96–19.21), MSD (19.21–52.23 ka), MSL (52.43–70 ka), SB22 (70–98.59), SB23 (99–103 ka). When individual stalagmite records were selected for the splice, it was aimed to achieve best possible age control and resolution. The data from Hulu Cave stalagmites have been corrected for a constant offset between the two caves^20^.

### Elemental records

**Figure 6:**
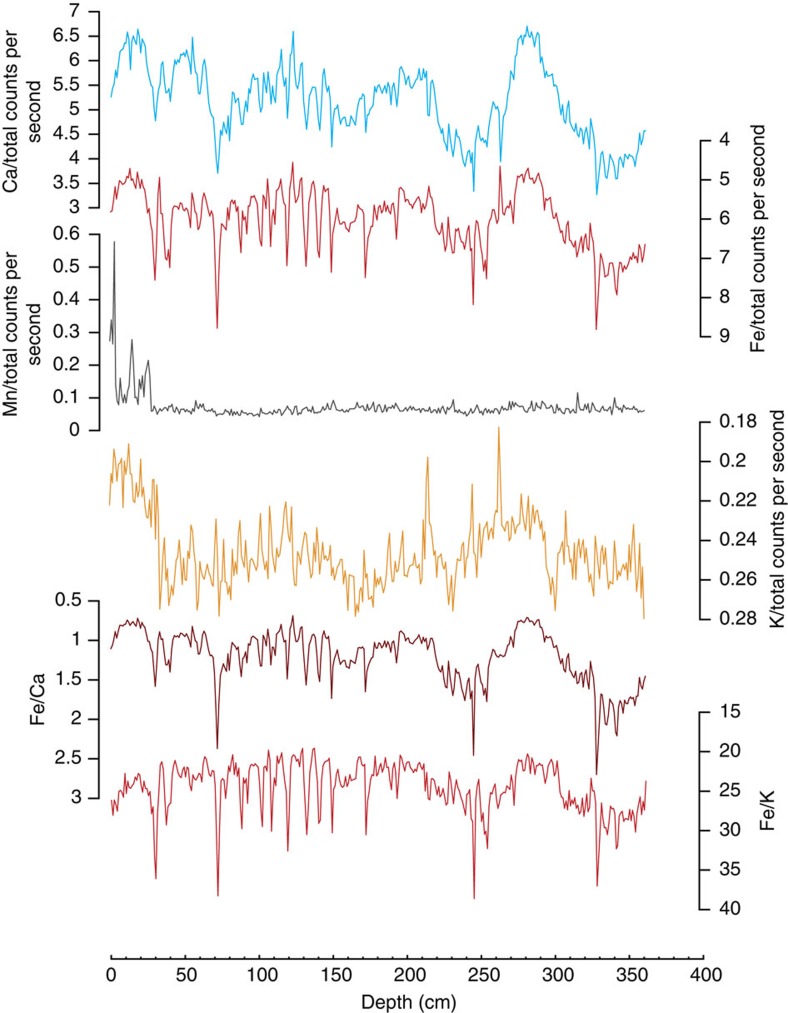
Additional XRF scanning profiles from CD154-17-17K. Ca normalized (Ca/total counts per second), Fe normalized, Mn normalized, K normalized, Fe/Ca and Fe/K ratios.

We use the variations in Fe and Fe/K ratios as proxies for terrestrial, fluvial input and indicators for humid/dry conditions on land, respectively. The XRF scanning intensities of terrestrial elements in marine sediment cores have been applied in many studies to trace the supply of terrestrial material to the ocean. Fe-XRF counts have been successfully applied in a large number of palaeoceanographic studies as proxy for varying river discharge in particular in tropical regions. The most proximal source for terrestrial material in the sediments of our core CD154-17-17K is the Great Kei river. Several other rivers also enter the Indian Ocean to the north of our core site. These include the Mbashe, Umzimvubu and Umtata rivers, as well as the Tugela; the largest in the KwaZulu-Natal Province. These rivers are all typical brown-water rivers, characterized by high sediment loads. Their sediments, in particular, those derived from the latosol-type soils, derive from mudstones and sandstones of the Karoo Supergroup and associated intruded dolerites (‘Ironstone') within the catchment areas, and are notably rich in iron oxides. Although sedimentary Fe can be prone to diagenetic remobilization in pore waters, we expect no diagenetic alteration of the Fe record due to the setting of CD154-17-17K in a low-productivity environment and with well-ventilated deep water bathing the site. Accordingly, downcore Fe concentration shows no correlation with Mn (Fig. 6), which also suggests that redox processes have no impact on the Fe record. Additionally, we also observe no oxide coatings on the biogenic carbonates. Dilution processes can also affect the interpretation of single-element concentrations. Carbonate dissolution and changes in carbonate production can substantially impact on single-element records. Being less sensitive to dilution effects, elemental ratios are more useful^60^. Fe is related to the siliciclastic components of the sediment and vary with the terrigenous fraction of the sediment, whereas Ca commonly reflects the biogenic carbonate content of the sediment. A detailed core-top study of in the Atlantic demonstrates that Fe/Ca ratios are very low on the mid-Atlantic ridge and high along the continental margins, in particular in regions of high fluvial input from the Gambia, Sanaga, Congo and Orange rivers along Africa, and from the Orinoco, Amazon and Plata rivers along South America^37^. In consequence of this distribution, Fe/Ca ratios have hence been used to trace changes in terrigenous input of mainly fluvial origin, particularly offshore Northeastern Brazil^61^,^62^, Western Africa^63^ as well as South-Eastern Africa^17^. Nevertheless, it needs to be noted that elemental ratios including Ca do not simply reflect the flux of terrigenous material, but they rather reflect the amount of terrigenous input relative to the biogenic carbonate flux/preservation.

## Author contributions

I.R.H. and R.Z. collected the core material. M.Z. and M.H.S. carried out the analytical work. M.Z. lead the writing of the manuscript. All authors contributed to interpretation of the data and writing the manuscript.

## Additional information

**How to cite this article:** Ziegler, M. *et al*. Development of Middle Stone Age innovation linked to rapid climate change. *Nat. Commun.* 4:1905 doi: 10.1038/ncomms2897 (2013).

## Figures and Tables

**Table 1:** ^14^C dates for CD154-17-17K.

**Depth (cm) MD02-2588**	**Species**	**14C age BP (years)**	**Error (years)**	**Calculated age, lower (years)**	**Calculated age, upper (years)**	**Code**
0–1	*G. ruber*	2,200	25	1,760	1,849	KIA 47083
18–19	*G. ruber*	6,735	+40/−35	7,220	7,314	KIA 47084
38–39	*G. ruber*	12,500	60	13,853	14,017	KIA 47085
48–49	*G. ruber*	16,120	90	18,702	18,941	KIA 47086
78–79	*G. ruber*	24,050	+200/−190	28,119	28,617	KIA 47087
126–127	*G. ruber*	35,340	+820/−720	39,071	40,881	KIA 47088
156–157	*G. ruber*	45,470	+3,080/−2,220	46,374	>50,000	KIA 47089
188–189	*G. ruber*	>44,780				KIA 47090

Calibration of radiocarbon dates was performed using the Calib software with the Marine09 calibration and Δ*R*=0. Calendar age range is 1*σ*.

**Table 2:** Age control points for the age model of sediment record CD154-17-17K.

**Radiocarbon dates**		**Tuning of planktonic** ***δ***^**18**^**O of CD154-17-17K to EPICA dome C on Speleo age**		**Additional tuning of Fe/K of CD154-17-17K to Chinese speleothem** ***δ***^**18**^**O splice**	
**Depth (cm)**	**Age (kyr)**	**Depth (cm)**	**Age (kyr)**	**Depth (cm)**	**Age (kyr)**
0.5	1.8	164.5	47	33.5	13.6
18.5	7.3	204.5	58.9	40.5	16.5
38.5	14.1	222.5	63.8	72.5	24
48.5	18.8	238.5	69.8	88.5	30.5
78.5	28.4	252.5	75.8	102.5	34.2
126.5	39.5	361.5	98	108.5	35.5
156.5	45.4			119.5	38.5
				126.5	39.5
				132.5	40.4
				141.5	42.1
				149.5	44
				156.5	45.4
				171.5	47.8
				178.5	49.3
				189.5	55.1
				193.5	55.7
				214.5	60.3
				242.5	71.2
				245.5	72.1
				255.5	77.1
				300.5	83
				326.5	90.5
